# Postharvest Preservation Strategies for Table Grapes: A Comprehensive Review from Practical Methods to Future Developments

**DOI:** 10.3390/plants14162462

**Published:** 2025-08-08

**Authors:** Ci Zhang, Qiankun Wang, Hui He, Yusen Wu, Wenpeng Shan, Hongru Liu

**Affiliations:** 1 Shanghai Academy of Agricultural Sciences, Shanghai 201403, China; cizhang6@saas.sh.cn (C.Z.);; 2 Shandong Academy of Grape, Shandong Academy of Agricultural Sciences, Jinan 250100, China; 3 Shanghai Institute of Ceramics, Chinese Academy of Sciences, Shanghai 200050, China

**Keywords:** grape preservation, postharvest storage, preservatives, controlled-atmosphere storage, biodegradable coatings, integrated technologies

## Abstract

Table grapes (fresh *Vitis vinifera* L. fruit) rank among the top five fruit crops worldwide, yet their high perishability poses significant challenges for postharvest handling and storage. This review offers a comprehensive analysis of current and emerging preservation strategies—including chemical fumigation, irradiation, packaging technologies, controlled-atmosphere (CA) storage, biodegradable coatings, and synergistic preservation systems. Distinct from prior studies that typically emphasize specific techniques or treatment categories, this work integrates mechanistic insights with technological advancements and industrial practices across multiple preservation modalities. It further evaluates the comparative effectiveness, limitations, and practical relevance of these strategies along the supply chain. Importantly, it identifies critical research gaps—such as the lack of cultivar-specific preservation protocols, the need for low-residue and environmentally sustainable treatments, and the absence of real-time quality monitoring systems. Addressing these gaps is essential for developing next-generation solutions. Finally, this review highlights practical implications by offering a forward-looking framework to guide innovation, providing grape producers and supply chain stakeholders with strategies to minimize losses, preserve quality, and enhance market competitiveness.

## 1. Introduction

Table grapes (fresh *Vitis vinifera* L. fruit) are among the world’s most important fruit crops. Grapes rank as the third most popular fruit globally after bananas and apples, with about 80 million tons produced annually [1]. Approximately 40% of this production (around 31.5 million tons) is destined for fresh consumption as table grapes [2]. The cultivation of table grapes spans over 50 countries, with China being the largest producer (12.6 million tons in 2022) and accounting for roughly 40% of global output [3]. Other major producing countries include India, Turkey, Egypt, and Iran, which together with China contribute nearly two-thirds of the world’s table grape supply. This fruit species is also remarkably rich in genetic diversity: more than 50 distinct table grape cultivars are known worldwide, encompassing a wide range of colors, flavors, and seed characteristics. Key international cultivars such as ‘Thompson Seedless’, ‘Red Globe’, and ‘Flame Seedless’ dominate many markets, while numerous region-specific varieties (e.g., ‘Barlinka’ in South Africa, ‘Muscat Hamburg’ in Australia) also play a significant role in local markets [4]. The broad cultivar base of table grapes underpins a rich assortment of quality traits and consumer preferences, but also means that shelf life and postharvest behavior can vary significantly among varieties. For example, the widely cultivated seedless variety Thompson Seedless tends to soften significantly during prolonged cold storage, whereas the newer variety NN107 retains firmer texture under identical conditions [5]. In contrast, Red Globe grapes grown in Sicily responded well to low-concentration SO_2_ fumigation, maintaining firmness, soluble solids, and acidity for up to three months while significantly reducing rachis and berry decay [6]. These examples highlight the importance of developing cultivar-specific preservation strategies to optimize postharvest outcomes.

As non-climacteric fruits, table grapes must be harvested at full ripeness to ensure optimal taste and nutritional quality. At harvest, they possess excellent organoleptic and nutritional qualities that drive high consumer demand [7]. Also, ripe grapes contain high levels of fermentable sugars (mainly glucose and fructose, often 15–20% by weight) which provide sweetness and energy [8]. They also contain essential minerals (such as potassium, calcium, and magnesium) and serve as a valuable source of vitamins, particularly vitamin C. In addition, grapes are packed with bioactive phytochemicals. Particularly in red and black cultivars, the skins and pulp are rich in polyphenolic compounds—including flavonoids, anthocyanins, and stilbenes (e.g., resveratrol)—which are well known for their antioxidant and health-promoting properties [9]. These functional constituents contribute to the acknowledged benefits of grape consumption (e.g., anti-inflammatory and cardioprotective effects) and enhance the fruit’s value as a nutritious food. Thus, as global populations and health-conscious diets continue to grow, the demand for fresh table grapes is expected to rise steadily in coming years.

Despite their popularity and nutritional richness, table grapes are highly perishable and prone to postharvest quality deterioration. The berries have thin skins and a high juice content, making them delicate and susceptible to mechanical injury and microbial infection. After harvest, grapes rapidly lose water through the skins and the stem attachment (rachis and pedicels), leading to rachis desiccation and browning, berry shriveling, weight loss, and loss of firmness [7]. Furthermore, the fruit is vulnerable to fungal decay, *Botrytis cinerea* in particular (causing “gray mold”) can spread quickly through grape clusters even at cold storage temperatures, resulting in significant spoilage [10,11,12]. These factors—dehydration and fungal spoilage—are the primary modes of quality degradation in table grapes [13,14,15].

Without proper postharvest handling, cumulative losses can be substantial: it is estimated that inadequate postharvest management can lead to 20–30% of table grapes being rendered unmarketable due to decay, shatter (berry drop), and other damage [16]. This high level of waste not only represents economic loss for producers but also undermines the availability of this nutritious fruit to consumers.

To combat these challenges, extensive research efforts have focused on improving the postharvest preservation of table grapes. Conventional approaches include sulfur dioxide (SO_2_) fumigation, cold storage combined with modified or controlled atmospheres, natural antimicrobial dips, physical treatments such as ultraviolet or electron-beam irradiation, and the use of advanced packaging materials or edible coatings to reduce water loss and decay [17,18].

Despite these advancements, quality deterioration and microbial spoilage remain significant, especially under extended storage or long-distance transport conditions. Meanwhile, regulatory restrictions on chemical residues and growing consumer demand for sustainable, low-impact preservation methods have further complicated grape postharvest management [19].

To address these issues, this review aims to identify critical knowledge gaps, consolidate recent advances, and explore emerging strategies—ranging from low-residue chemical alternatives to smart packaging technologies. Unlike previous reviews, we place emphasis on cross-disciplinary integration and future-oriented solutions to guide the development of more sustainable, effective, and scalable postharvest systems for table grapes.

## 2. Postharvest Preservation Technologies

Maintaining the postharvest quality and safety of table grapes necessitates a variety of preservation strategies, each designed to address specific issues such as microbial spoilage, water loss, and physiological degradation. This section provides an overview of key chemical, physical, and integrated approaches, emphasizing their underlying mechanisms, effectiveness, and practical applications. Major technological advancements are reviewed, including sulfur dioxide (SO_2_) fumigation, irradiation, liquid-based treatments, innovative packaging solutions, modified atmosphere storage, biodegradable coatings, and combined preservation systems (Table 1).

**Figure 1:**
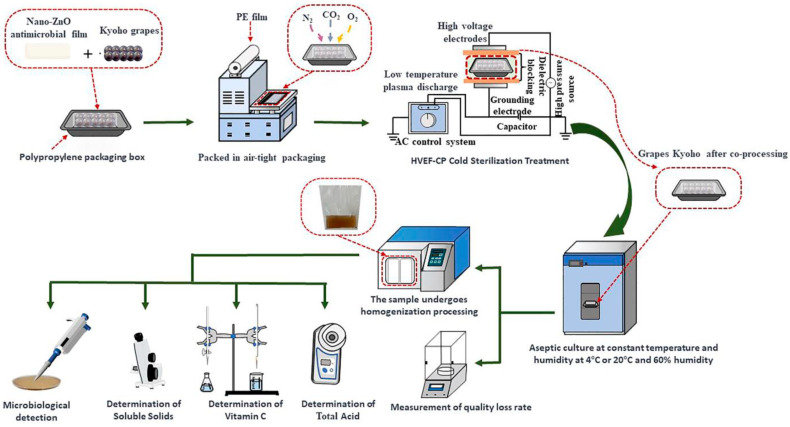
Schematic diagram showing the stepwise process of grape treatment and quality assessment using HVEF-CP and nano-ZnO antimicrobial film. Reproduced with permission [20], published under the CC BY license.

To effectively implement these technologies and ensure their reliability, it is equally important to establish standardized evaluation protocols. A typical assessment involves microbiological analysis, physicochemical property testing, and quality change monitoring under defined storage conditions. These standardized protocols ensure the reliability and comparability of results across different studies. Process flowcharts are often used to illustrate comprehensive workflows—from sample preparation to data collection—and support reproducibility. For example, a schematic involving nano-ZnO antimicrobial films and high-voltage electric field-assisted cold plasma (HVEF-CP) treatment outlines the sequential steps of grape selection, inoculation, packaging, treatment, storage, and evaluation (Figure 1). Such integration of advanced techniques with standardized assessments provides a model for future research in grape preservation [20].

**Table 1:** Overview of postharvest preservation technologies for table grapes: mechanisms, advantages, limitations, and references.

Preservation Technologies	Mechanism	Key Parameters	Advantages	Limitations	References
SO_2_ fumigation	Inhibits microbial growth and oxidation via sulfur dioxide release	Fumigant concentration; exposure time; storage temperature	Highly effective against gray mold; low cost; long-standing commercial use	Potential residue issues; consumer sensitivity; regulatory restrictions	[17,18,21,22,23,24,25,26,27,28,29]
Irradiation	Destroys microorganisms and pests with ionizing radiation	Radiation dose; exposure duration	Non-thermal; broad-spectrum efficacy; can replace chemical fumigants	Expensive equipment; possible changes in texture/flavor; consumer perception issues	[30,31,32,33,34,35,36,37,38,39,40,41,42,43,44,45,46,47,48]
Liquid treatments	Creates antimicrobial environment using agents like ethanol, chitosan, etc.	Type of liquid; concentration; application method; treatment duration; temperature	Simple application (spray/dip); low toxicity; some materials are natural and biodegradable	Shorter-lasting effect; requires direct contact; excess moisture may cause decay	[49,50,51,52,53,54,55,56,57,58,59,60,61,62,63,64,65,66,67,68]
Sealing and packaging	Blocks air, moisture, and contaminants	Packaging material type; barrier properties; sealing conditions, storage temperature	Prevents dehydration and mechanical damage; maintains appearance during transport	Passive protection; limited control over internal microenvironment	[69,70]
Modified atmosphere packaging	Alters O_2_/CO_2_ levels to reduce respiration and spoilage	Gas composition; permeability of packaging film; temperature; storage duration	Delays senescence; suppresses pathogens; prolongs shelf life; suitable for export	Requires precise gas composition control; potential flavor loss; cost of active systems	[71,72,73,74,75,76,77,78,79,80,81,82]
Biodegradable/Edible coatings	Forms a physical and biochemical barrier on grape surface	Film composition; coating thickness; bioactive additives; drying conditions; storage temperature	Environmentally friendly; reduces water loss; can incorporate functional agents (e.g., essential oils)	Mechanical fragility; inconsistent coating uniformity; short shelf life without cold chain	[83,84,85,86,87,88,89]
Synergistic technologies	Integrates multiple methods (e.g., MAP + ethanol; UV-C + chitosan)	Type and sequence of methods; compatibility; application timing; storage environment	Combines strengths of each technique; enhances efficacy and spectrum; lowers chemical dependency	May require optimization for specific cultivars; increased system complexity and cost	[90,91,92,93,94,95]

### 2.1. SO_2_ Fumigation

**Figure 2:**
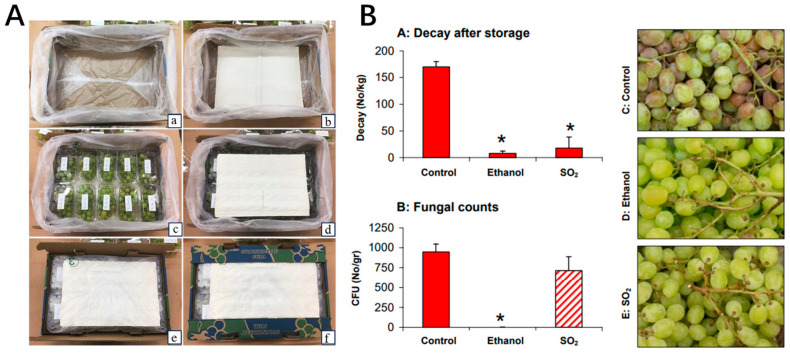
(**A**) Packaging steps of table grape. (**a**) A carton box lined with perforated plastic liners; (**b**) A sheet of moisture-absorbing paper placed at the bottom of the box; (**c**) Vented plastic clamshells containing grapes are arranged inside the box; (**d**) An SO_2_-generating is positioned on top of the clamshells; (**e**) The plastic liners are sealed using adhesive tape; (**f**) The fully packed carton is now ready for placement in cold storage. Reproduced with permission [24]. Published under the CC BY license. (**B**) Decay and fungal counts of ‘Early Sweet’ grapes after ethanol dip or SO_2_ fumigation. * indicates significant differences from the control. Reproduced with permission [17]. Copyright 2006, Stewart Postharvest Solutions (UK) Ltd.

Sulfur dioxide (SO_2_) fumigation is one of the most widely used and effective methods for controlling postharvest fungal diseases, particularly gray mold (*Botrytis cinerea*) [21]. SO_2_ not only prevents microbial spoilage but also regulates sugar metabolism, phenylpropanoid pathways, and aroma synthesis in grapes [22]. Application typically involves fumigation chambers or controlled-release pads during cold storage [23]. The general processing steps for SO_2_ fumigation include grape selection and packaging, placement of SO_2_-generating pads (or exposure to gaseous SO_2_), cold storage at controlled temperature (0–1 °C), and periodic ventilation to avoid excessive residue (Figure 2A) [24]. In commercial practice, SO_2_ generator pads, typically containing 5–7 g of active compound, are widely used in grape packaging for their simplicity and sustained antimicrobial action. Alternative forms include gas fumigation and slow-release sachets, each offering varying control over dosage and release rate to meet different grape varieties and storage needs [18,25,26,27]. As shown in Figure 2B, compared to the control, ‘Early Sweet’ grapes treated with SO_2_ generator pads had significantly fewer decayed berries after 35 days of cold storage plus 3 days at 20 °C. Fungal counts before storage show that ethanol dipping reduced the initial fungal load, while SO_2_, which activates during storage, eliminated fungal growth in follow-up experiments. Visual comparison shows that SO_2_-treated grapes remained in better condition than untreated or ethanol-treated ones, confirming the treatment’s strong preservative effect during storage [17].

However, excessive SO_2_ exposure can result in rachis browning, berry cracking or detachment, and potential allergic reactions in sensitive individuals [28,29]. While SO_2_ remains critical due to its antimicrobial, antioxidant, and anti-respiratory properties, optimal dosage strategies must balance efficacy with food safety and sensory quality. Further studies are needed to fine-tune treatment parameters across different grape cultivars to achieve low-residue outcomes.

### 2.2. Irradiation

Irradiation is a non-thermal sterilization method utilizing gamma rays, electron beams, or X-rays to eliminate pathogens by inducing DNA damage or generating reactive oxygen species. Its application in table grapes has demonstrated impressive outcomes in reducing microbial load and extending storage life [30,31,32,33,34,35,36,37]. By ionizing the small molecules in water and microbial cells, it generates active free radicals or directly destroys the DNA of microbial cells, thus achieving the sterilization effect [38,39,40]. The general irradiation treatment process involves grape sorting and packaging and irradiation at a defined dose (e.g., 0.2–1.0 kGy) under controlled conditions, followed by cold storage and post-treatment evaluation of microbial and physicochemical parameters. Irradiation preservation technology has shown great potential in grape preservation, with various studies exploring its effects on different aspects of grape quality and storage. Electron beam irradiation effectively reduced microbial load and decay rate in imported U.S. table grapes, with 0.56 kGy identified as the optimal dose for preservation [41]. The combination of high-energy electron beam irradiation with SO_2_ treatment demonstrated enhanced efficacy in controlling microbial growth, reducing respiration rate, maintaining titratable acidity and vitamin C content, and preserving duperoxide dismutase activity in ‘Kyoho’ grapes [30]. Moreover, the Kyoho grape could optimally maintain its physiological quality and freshness under 0.7 kGy irradiation. Compared with the control group, the storage quality was significantly improved when preserved for 98 days at −0.5 °C [42]. Research has also been conducted on other grape-related preservation scenarios. The suitable dose of ^60^Co-γ radiation on grape cuttings and fruits was determined to be 0.6 kGy. This dose effectively inhibited the occurrence and expansion of grape diseases, thereby reducing the use of chemical fungicides [43]. UV-C treatment also demonstrated its positive impact on grape quality. It significantly boosted phenolic content and antioxidant activity in grapes during the first 14 days of storage, outperforming both the control and UV-B treatment [44]. Furthermore, the application of irradiation in the wine industry has been explored. A study found that X-ray and electron beam irradiation of grapes before winemaking significantly enhances the extraction of phenolic and aromatic compounds, revealing the great potential of irradiation technology in the wine industry [45]. Another interesting finding was that the preservation effect of 1-MCP combined with ^60^Co-γ irradiation treatment is better than that of 1-MCP treatment alone. This combination could significantly delay the aging process of crystal grapes, providing new ideas and a reference basis for the storage and preservation of crystal grapes [46].

It is important to note that combining irradiation with complementary technologies amplifies the preservation efficacy. However, despite its numerous advantages, irradiation also faces practical limitations. These include equipment costs and consumer perception [47,48]. To further expand the application and effectiveness of irradiation in grape preservation, future research should focus on optimizing dose–response relationships for different grape varieties. This will ensure that the most appropriate irradiation dosage can be determined for each specific variety to achieve the best preservation results. Additionally, developing real-time quality monitoring systems is crucial. Such systems will help ensure regulatory compliance by continuously monitoring the quality of grapes during storage and irradiation processes.

### 2.3. Preservation Methods Using Liquids: Spraying, Vaporing, and Immersing

Liquid-based preservation techniques—including spraying, vaporizing, and immersing—play a critical role in extending the shelf life and maintaining the postharvest quality of table grapes. Each method delivers preservation agents through different mechanisms, offering flexibility based on grape variety, logistics, and preservation goals. Spraying primarily targets the fruit surface and triggers localized defense responses [49]. Vaporizing enables volatile compounds to penetrate tissues, influencing internal metabolic pathways and enzyme activity [50]. Immersion allows more uniform contact and deeper penetration, leading to stronger physiological effects [51]. These differences allow each method to exert distinct regulatory impacts on grape defense and quality, enabling tailored preservation strategies.

Spraying involves directly applying liquid formulations onto grape surfaces and is favored for its simplicity and broad coverage. For instance, grape clusters treated with *Aloe vera* gel one day before harvest and stored at 2 °C for 35 days exhibited only 1% decay compared to 15% in untreated controls [52]. Similarly, when sprayed preharvest and applied as a coating postharvest, chitosan significantly reduced decay over 42 days of storage at 0 °C, lowering the decay index from 0.15 to 0.05 [53]. Moreover, integrating preharvest spray with *C. laurentii* and postharvest chitosan coating treatment has proven effective for both the decay control and quality maintenance of table grapes [54,55].

Vaporizing utilizes gaseous preservatives to delay ripening and suppress microbial growth without direct liquid contact. Studies have evaluated using acetic acid (AC) vapors to control *Botrytis cinerea* in table grapes, finding it effective in reducing gray mold incidence compared to untreated grapes, with repeated treatments being most effective [56,57]. Ethanol vapor treatment at 2 mL kg^−1^ optimized over two seasons prevented rot and stem browning in ‘Chasselas’ grapes as effectively as sulfur dioxide pads, with no significant sensory difference detected by consumers; the method could be easily adopted by the industry due to its similarity to sulfur dioxide treatment technology [58].

Immersion allows for uniform preservative distribution by submerging grapes in liquid solutions. When Redglobe grapes were immersed in chitosan and stored for 4 weeks at 0–1 °C, there were 10 infected berries per kg, compared to 19 infected berries in the control group. This indicates that chitosan treatment significantly reduced the incidence of infection in the stored grapes [59]. Exposure to 30% ethanol for 10 s at 24 °C has been found to inhibit *Botrytis cinerea* spore germination. Immersing infected grapes in 30% ethanol reduces decay by about 50% after 35 days of storage at 1 °C. Furthermore, ethanol enhances the efficacy of hot water treatments for grapes inoculated with *B. cinerea*. Prompt drying is important to prevent berry cracking [60]. A related study found that briefly immersing grape berries in water or ethanol at different temperatures can reduce the incidence of gray mold caused by *Botrytis cinerea*, with heated ethanol showing better effectiveness [61]. Ethanol dipping is another form of immersing that has shown great promise. Among the various technologies proposed, ethanol dipping combined with a modified atmosphere (MA) appears most suitable for ready-to-eat grapes. This combination addresses both stringent sanitation demands and protection against post-treatment contamination [62,63].

Essential oils and extracts have recently gained significant attention in the field of grape preservation due to their antibacterial, antifungal, antioxidant, and bio-regulatory properties [64]. Essential oils are concentrated hydrophobic liquids containing volatile aromatic compounds extracted from plants. The volatile components of essential oils are typically classified into four main categories: terpenes, benzene derivatives, hydrocarbons, and other miscellaneous compounds. Essential oils and plant extracts from various plants have been found to exhibit biological activity, offering insecticidal, antimicrobial, antioxidant, and bio-regulatory properties [65]. Currently, around 3000 essential oils are known, with 300 being commercially valuable, particularly in industries such as pharmaceuticals, agriculture, food, sanitation, cosmetics, and fragrance [18]. Sukatta et al. highlighted the effectiveness of clove and cinnamon oils in controlling postharvest decay in grapes caused by six fungal species, including *Aspergillus*, *Alternaria*, *Colletotrichum*, *Lasiodiplodia*, *Phomopsis*, and *Rhizopus*. The study showed that cinnamon oil had stronger antifungal effects than clove oil. Their combinations at higher cinnamon ratios (3:7, 2:8, 1:9) inhibited all six fungi at 400 mg/mL, highlighting their synergistic potential for postharvest grape preservation [66]. Further studies by other researchers have also shown that cinnamon and clove oils are highly effective antifungal agents for controlling various table grape diseases [67,68].

In summary, liquid-based preservation methods—whether utilizing natural products like *Aloe vera*, chitosan, ethanol, or plant essential oils—offer diverse, adaptable approaches to enhance grape storage outcomes. Each technique brings unique advantages in terms of efficacy, application ease, and compatibility with postharvest handling, making them essential components of integrated preservation strategies.

### 2.4. Sealing and Packaging

Effective sealing and packaging technologies are essential for reducing postharvest losses in table grapes, which are prone to microbial spoilage, water loss, and oxidative deterioration. Recent advances in packaging design have significantly improved grape preservation by creating controlled microenvironments that maintain fruit quality during storage and transport. Modern packaging strategies—including modified atmosphere packaging (MAP), vacuum packaging (VP), and active packaging systems—have revolutionized grape storage. These technologies regulate gas exchange, moisture retention, and microbial growth, thereby extending shelf life while preserving sensory and nutritional properties.

A study evaluating the postharvest quality of fresh-cut grapes stored at 5 °C over 14 days demonstrated the importance of the packaging material and cutting method. Among the tested conditions, four-berry clusters packed in PVC bags showed the lowest weight loss, minimal decay, and reduced berry shattering, receiving the highest consumer acceptability ratings [69]. Packaging systems can also be enhanced by combining them with other preservation methods. For instance, the application of hot water treatment and retention of cap stems significantly reduced microbial contamination and decay in packaged grapes under refrigerated conditions. Importantly, these treatments did not adversely affect grape texture, color, or flavor, indicating their compatibility with high-quality postharvest management [70].

Together, these findings underscore the pivotal role of packaging not only as a passive barrier but also as an active component in integrated preservation systems. Continued innovation in packaging materials and combined treatment protocols holds great promise for further improving the storage stability and marketability of table grapes.

### 2.5. Modified Atmosphere Packaging

**Figure 3:**
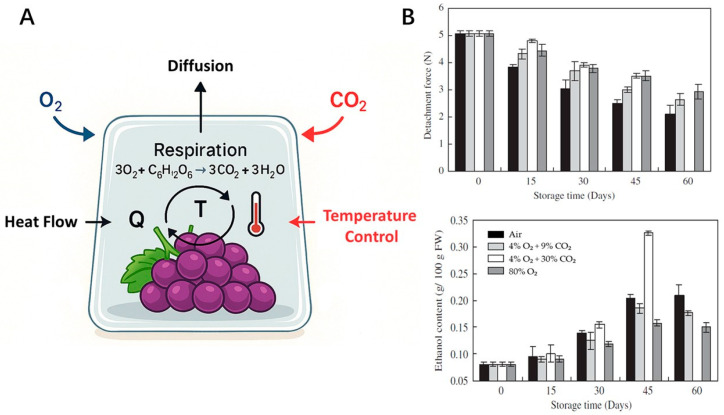
(**A**) Schematic illustration showing the MAP system for table grapes. (**B**) Ethanol content and detachment force of table grapes stored under different atmospheric conditions (Air, 4% O_2_ + 9% CO_2_, 4% O_2_ + 30% CO_2_, and 80% O_2_) at 0 °C for 60 days. Reproduced with permission [76]. Copyright 2006, Elsevier Ltd.

Modified atmosphere packaging (MAP) is an advanced storage technology designed to extend the shelf life of fruits by adjusting the internal gas composition [71]. By optimizing O_2_ and CO_2_ levels, MAP slows down respiration rates, reduces ethylene production, and mitigates physiological or pathological deterioration (Figure 3A). This controlled microenvironment helps maintain fruit freshness, texture, and nutritional quality during storage and transportation. For clarity, the typical steps involved in MAP application include grape cleaning and sorting, packaging in gas-permeable films, adjustment of internal atmosphere (e.g., lowering O_2_ and increasing CO_2_), sealing, and cold storage. These procedures ensure extended shelf life through the synergistic control of microbial growth and physiological decay.

Advanced MAP systems with high CO_2_ can effectively maintain quality and extend the shelf lives of fruits and vegetables [72]. High-CO_2_ atmospheres offer a viable SO_2_ alternative for *Botrytis* control in ‘Redglobe’ grapes, although precise CO_2_/O_2_ balancing is essential to mitigate sensory penalties and extend shelf life [73]. Research has identified optimal gas compositions for specific fruits. A 10% CO_2_ concentration has emerged as ideal for MA storage, as a higher level can impair grape flavor, while lower levels fail to suppress decay effectively [17]. Artes-Hernandez et al. demonstrated that ‘Autumn Seedless’ grapes could be successfully stored in MAP for up to 60 days while maintaining acceptable quality [74]. This approach significantly reduced the decay rate from 8.6% to 2.6%, enhancing the fruit’s overall shelf life and quality. However, not all MAP systems are equally effective. A study comparing passive and active MAP systems found that while all packaging materials significantly extended shelf life compared to unpackaged grapes, active MAP was less effective due to rapid gas equilibrium and increased dehydration [75]. High-oxygen (O_2_) treatment has also gained attention as a practical method to prevent enzymatic discoloration, anaerobic fermentation, and the growth of aerobic and anaerobic microbes. High oxygen (80% O_2_) has shown promise as a long-term preservation method for “Kyoho” grapes. While high CO_2_ is effective in the short term, storage periods must be strictly limited to less than 45 days (Figure 3B). Future research should focus on optimizing the safety of high-oxygen environments and controlling costs to promote its application in organic and high-end fruit and vegetable markets [76].

Beyond conventional MAP that regulates O_2_ and CO_2_ levels, ozone-enriched atmospheres have emerged as an effective extension of MAP strategies. Ozone can be introduced in gaseous or aqueous form and decomposes into reactive oxygen species (e.g., hydroxyl radicals) that disrupt microbial membranes and inhibit pathogen growth. This approach not only enhances microbial safety but also helps preserve the sensory and nutritional quality of grapes during storage. As a part of MAP-based interventions, ozone treatment provides a promising avenue for reducing chemical residues while maintaining postharvest quality [77]. Despite its potential, the application of ozone requires careful control, as the effective concentration for pathogen inhibition is close to the threshold that may cause adverse effects on the fruit [17]. Ozone can also be applied as a short-term pre-storage method in air or water or incorporated into the storage atmosphere either continuously or intermittently [78,79]. Studies have shown that ozone treatment during cold storage effectively reduces decay in table grapes, with continuous ozone being more effective than intermittent ozone. Interestingly, intermittent ozone treatment increased the resveratrol content in grapes, suggesting its potential to enhance nutritional value [80]. Additionally, ozone in water at 10 µg/mL significantly controlled gray mold on grapes [81].

Looking ahead, innovations in smart packaging, such as pH-responsive or temperature-sensitive films, aim to dynamically adjust the microenvironment during storage. These advancements could be combined with the integration of probiotics or plant-derived antimicrobial peptides into packaging, offering the dual functionalities of safety enhancement and health promotion. Furthermore, studies have explored the potential of enhancing grape flavor by storing berries in atmospheres enriched with monoterpenes, such as linalool or geraniol [82]. These findings suggest that using monoterpenes during storage can improve grape flavor, adding another dimension to the application of modified atmospheres in postharvest management

Overall, MAP and its advanced derivatives offer versatile, non-chemical solutions for maintaining grape quality throughout the postharvest supply chain. Continued research into gas composition optimization, additive integration, and real-time atmosphere monitoring will further expand the utility of MAP in high-value fruit preservation.

### 2.6. Biodegradable or Edible Coating

The need for sustainable and effective food preservation methods has driven significant research into biodegradable, renewable, and non-polluting biopolymers as alternatives to traditional petroleum-based packaging materials. Materials such as gelatin (Gn) and polyvinyl alcohol (PVA) have gained attention due to their biodegradability and environmental friendliness. However, these biopolymers often lack sufficient mechanical strength and antimicrobial properties, limiting their practical applications. To address these limitations, researchers have developed composite films by incorporating various nanoparticles and bioactive compounds into biopolymer matrices. Kehui et al. demonstrated the potential of incorporating ZnO@QAC NPs into PVA/Gn matrices to create composite films with enhanced properties [83]. When used to wrap grapes, films containing 2% ZnO significantly reduced weight loss, with a 40.13% decrease observed after 15 days compared to unwrapped grapes. This highlights the excellent preservation efficacy of these composite films in maintaining grape quality during storage. Similarly, Mahnaz explored the use of edible films incorporating *Dracocephalum kotschyi* (*D. kotschyi*) essential oil nanoemulsion into a chitosan–gelatin polymeric matrix for grape preservation at room temperature [84]. The films containing 5% essential oil exhibited strong antibacterial activity against specific pathogens. Moreover, SEM analysis confirmed that the incorporation of the nanoemulsion led to the formation of microporous structures in the films. Chitosan, particularly in its dissolved form in acetic acid, has also shown significant potential in controlling postharvest decay. Studies have demonstrated its effectiveness in preserving both single berries stored at 15 °C and small clusters stored at 0 °C for 60 days [85].

In addition to chitosan, another natural approach involves the utilization of an *Aloe vera* gel coating [86], which has proven effective in both preharvest and postharvest applications for controlling postharvest gray mold in table grapes. Boron, in the form of potassium tetraborate strongly inhibited spore germination, germ tube elongation, and mycelial spread of *B. cinerea* in the culture medium [87]. A study found that the essential oils from sweet basil, fennel, summer savory, and thyme effectively inhibited the mycelial growth of *B. cinerea*, a major cause of postharvest losses in table grapes. The results suggest that essential oils, especially those rich in phenolic compounds, could serve as safe and beneficial tools for preserving table grapes [88]. Another innovative approach involves the use of Citronella oil (CO) incorporated into soy protein isolate (SPI) to create antimicrobial protein films. These CO-containing SPI films exhibit strong potential for extending the shelf life of fresh produce. Notably, the film with 3% CO reduced grape weight loss to 44.16% after 7 days of storage, demonstrating its superior water-retention performance and applicability in food preservation [89] However, the incorporation of CO significantly mitigated this loss, particularly during the final three days of storage. On Day 1, the weight loss for 0%, 1%, 2%, and 3% CO films was 13.88%, 12.97%, 13.71%, and 11.98%, respectively. By Day 7, these values rose to 54.42%, 49.14%, 47.50%, and 44.16%. This improvement is attributed to the hydrophobic nature of CO, which condenses water vapor on the film surface, thereby reducing moisture evaporation and maintaining grape quality during storage.

In summary, biodegradable and edible coatings—especially those enhanced with nanoparticles or essential oils—offer multifunctional protection by combining physical barriers, antimicrobial effects, and environmental sustainability. Their integration into postharvest management systems represents a critical step toward greener, safer, and more efficient grape preservation solutions.

### 2.7. Synergistic Effects with Emerging Technologies

Integrating various preservation methods can effectively address the inherent limitations of individual approaches, thereby enhancing the overall prevention of spoilage in table grapes during storage. These combined strategies not only improve shelf life but also maintain fruit quality and safety through complementary mechanisms. This section highlights several innovative combinations of preservation techniques that have shown promising results in extending the shelf life and improving the postharvest performance of table grapes. One innovative example involves the development of an active packaging system infused with natural antimicrobial agents—such as eugenol, thymol, and menthol—combined with MAP. This combined approach offers synergistic benefits: MAP slows physiological deterioration, while the antimicrobials inhibit microbial spoilage. Together, they significantly reduced decay incidence and maintained grape quality, extending shelf life by approximately three weeks compared to conventional MAP alone [90]. However, while these plant-derived compounds are effective against a broad spectrum of microorganisms, their strong aromas may negatively impact consumer sensory perception, particularly when concentrations are not carefully controlled. Additionally, the high cost of some essential oils can hinder scalability in commercial settings. Ethanol-based treatments, when used in conjunction with MAP, have also shown strong synergistic effects. Ethanol vapor effectively controlled gray mold without damaging berries, matching or exceeding the efficacy of SO_2_ generator pads. Furthermore, any off-flavors introduced during storage were eliminated simply by airing the grapes for one day prior to consumption. This mild and continuous preservation method offers a practical and safe alternative to traditional fumigation [62,91,92]. Ethanol vapor’s compatibility with MAP systems enables extended application duration without the need for repeated manual treatments, making it suitable for cold-chain logistics. Combining chitosan coatings with UV-C radiation has proven particularly effective in controlling *B. cinerea* on certain grape varieties. This dual treatment not only forms a physical barrier but also triggers host defense responses, including increased production of catechins, resveratrol, and chitinase enzymes—key components of the grape’s natural resistance mechanisms [93]. Importantly, this combination offers a chemical-free approach to preservation, addressing concerns about residue and regulatory compliance. Another promising strategy involves combining ethanol with potassium sorbate. This combination effectively inhibits *B. cinerea* spore germination and reduces gray mold incidence on grapes. It showed better efficacy than either treatment alone and matched the performance of commercial SO_2_ pads, without causing berry damage [94]. Furthermore, the integration of calcium chloride (CaCl_2_) with MAP has demonstrated synergistic benefits. In a study by Imlak, grapes pretreated with 2% CaCl_2_ and stored under a 5% CO_2_ atmosphere maintained optimal firmness, acidity, and phenolic content, while also minimizing increases in soluble solids. This treatment also significantly reduced browning caused by gray mold compared to the water-washed control [95]. Calcium plays a well-known role in maintaining cell wall integrity and delaying senescence, thus enhancing the effectiveness of atmospheric modifications.

Overall, the success of these synergistic systems lies in their ability to combine complementary modes of action—such as antimicrobial, antioxidant, physical barrier formation, and physiological modulation—into unified preservation solutions. Future research should focus on optimizing formulation ratios, assessing economic feasibility, and developing targeted combinations based on grape cultivar, market demand, and regulatory constraints.

## 3. Toward Next-Generation Preservation Technologies

With the continuous advancement of science and technology, fruit and vegetable preservation technologies have become increasingly diversified and refined [96]. Postharvest losses remain significant, with global estimates reaching up to one-third of total production, leading to major economic impacts [97]. Meanwhile, growing public concerns about food safety have driven demand for low-dose, low-sulfur, and environmentally friendly preservation methods [24,98]. These approaches aim to decrease chemical residues while ensuring quality and safety. In this context, both chemical and physical preservation methods have gained significant attention due to their ease of operation, extended efficacy, and technical maturity.

Currently, treatments such as SO_2_ and ClO_2_ are widely used and have proven effective in prolonging grape shelf life and controlling *B. cinerea*. However, their effectiveness depends heavily on precise dosage control—excessive concentrations can lead to chemical residues or undesirable bleaching. Conversely, alternatives like 1-MCP, sec-butylamine, ethanol, calcium peroxide, and hydrogen sulfide present lower bleaching risk, but often fall short in antimicrobial efficacy. Physical preservation techniques—though residue-free and clean—generally offer limited protective effect when used alone, especially in comparison to SO_2_-based systems.

To address these limitations, future research should prioritize the development of preservation methods that are residue-free, dosage-controllable, and non-resistance-inducing. A promising path lies in synergistic systems that combine physical and chemical techniques to accommodate the physiological diversity of different grape cultivars. In this regard, interdisciplinary innovation—integrating food science, polymer engineering, nanotechnology, microbiology, AI-driven quality monitoring, and blockchain-enabled supply chain transparency—will be essential.

**Figure 4:**
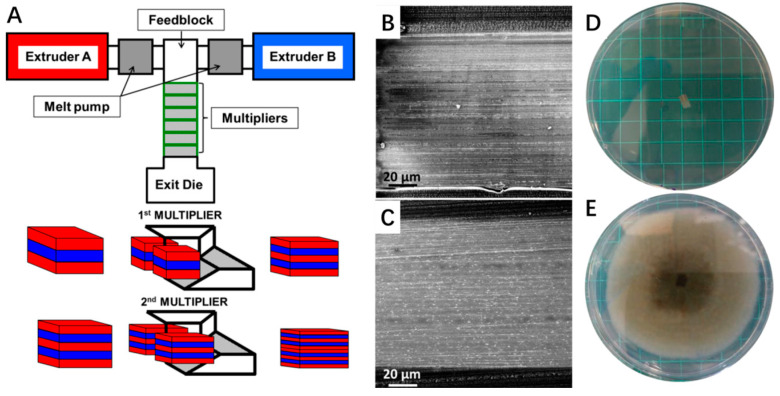
Antimicrobial multilayered film design, morphology, and bioactivity. (**A**) Schematic illustration of the co-extrusion and multiplication process used to fabricate multilayered films. (**B**,**C**) Cross-sectional morphology of 65-layer films: (**B**) (LDPE/carvacrol)/EVOH and (**C**) (LDPE/[HNTs/carvacrol])/EVOH. (**D**,**E**) Antifungal activity of the films against *A. alternata*: (**D**) inhibition by carvacrol-containing multilayered films; (**E**) unhindered fungal growth observed with a 9-layer LDPE/EVOH control film. Reproduced with permission [99], published under the CC BY license.

A particularly promising direction involves the development of multilayered antimicrobial polymer films, which allow for the controlled release of natural preservatives such as essential oils. For instance, recent studies have demonstrated the successful fabrication of multilayer films containing carvacrol—a natural antimicrobial compound—via microlayer co-extrusion techniques [99]. These films, composed of alternating layers of LDPE and EVOH, retained a high carvacrol content despite harsh processing conditions (200 °C) and exhibited significantly reduced oxygen permeability and volatile diffusion compared to conventional single-layer films (Figure 4). The inclusion of halloysite nanotubes (HNTs) as carriers further enhanced carvacrol’s thermal stability and sustained release performance. The resulting films exhibited robust antibacterial and antifungal efficacy in both in vitro assays and real-food models (e.g., cherry tomatoes), without compromising material integrity.

This multilayer packaging strategy represents a robust and flexible platform for embedding sensitive bioactive agents into commercial packaging materials, enabling precision-controlled, residue-free, and highly effective preservation. However, to transition from lab-scale innovation to widespread commercial adoption, further efforts must be directed toward scaling up production, reducing costs, and ensuring compatibility with existing cold-chain logistics and packaging infrastructures. As such, the integration of smart multilayer packaging—especially packing loaded with natural antimicrobials and nanocarriers—represents a promising frontier for next-generation grape preservation systems.

Beyond material innovations, future grape preservation systems must also be adapted to diverse and often resource-constrained storage scenarios. For instance, in regions lacking cold-chain infrastructure or in smallholder-based supply chains, traditional approaches may be impractical. Alternative solutions—such as solar-powered storage, low-tech biodegradable packaging, or vapor-phase essential oils—are being explored for their feasibility and sustainability in these decentralized settings [100].

**Figure 5:**
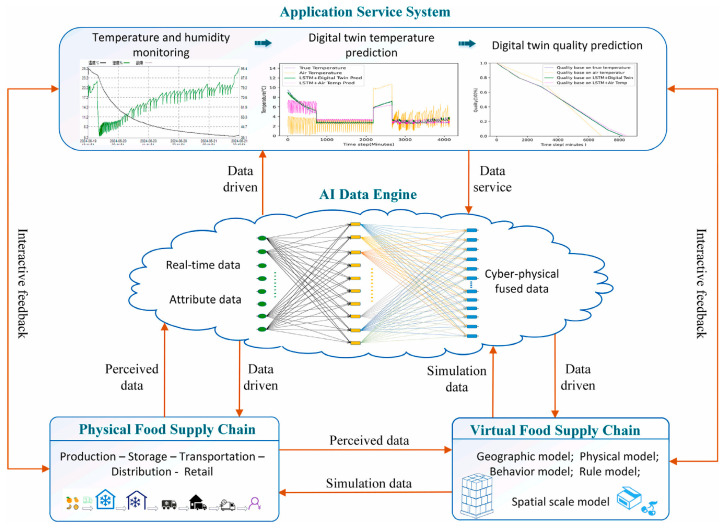
Digital twin and AI model-based cold supply chain. Reproduced with permission [102], published under the CC BY license.

Concurrently, artificial intelligence (AI) is poised to transform grape preservation by enabling dynamic, data-driven decision-making. AI-powered digital twin models simulate different storage or transport scenarios, allowing preemptive adjustments to avoid losses [101]. In-package sensors combined with machine learning can detect volatile signatures, temperature drift, or visual signs of decay—triggering responsive preservation actions such as gas regulation or antimicrobial release (Figure 5) [102]. These smart preservation systems optimize shelf life while reducing waste and labor, and have demonstrated promising results in both lab and real-world settings. Moreover, AI-based freshness prediction models can forecast shelf life from hyperspectral or time-series data, enabling real-time inventory routing, pricing decisions, and traceability across supply chains [103,104].

Together, the convergence of adaptive packaging and AI-enabled monitoring presents a multidimensional framework for next-generation grape preservation—one that is precise, sustainable, and responsive to both biological and logistical complexity.

## 4. Conclusions

Achieving effective postharvest preservation of table grapes requires a careful balance between maintaining fruit quality, ensuring food safety, and promoting environmental sustainability. This review has examined a broad spectrum of preservation strategies—chemical, physical, and biological—with increasing attention to natural antimicrobials, nanotechnology-based films, and intelligent packaging systems. Notably, the success of these methods often hinges on the delivery approach, underscoring the importance of context-specific application. Looking ahead, future preservation systems are expected to evolve toward integrated, low-residue, and precision-controlled solutions tailored to specific cultivars and supply chain conditions. The integration of digital innovations—such as AI-powered monitoring and blockchain-enabled traceability—offers promising avenues to enhance real-time quality control and supply chain transparency. Ultimately, advancing grape preservation will depend on sustained interdisciplinary collaboration, field-scale validation, and the development of scalable, cost-effective, and eco-friendly technologies suited for commercial deployment.

## Data Availability

No new data were created or analyzed in this study. Data sharing is not applicable in this study.
